# Variation in geographic access to chemotherapy by definitions of providers and service locations: a population-based observational study

**DOI:** 10.1186/s12913-016-1549-5

**Published:** 2016-07-18

**Authors:** Mary C. Schroeder, Cole G. Chapman, Matthew C. Nattinger, Thorvardur R. Halfdanarson, Taher Abu-Hejleh, Yu-Yu Tien, John M. Brooks

**Affiliations:** Department of Pharmacy Practice and Science, College of Pharmacy, University of Iowa, 115 South Grand Ave, S525 PHAR, Iowa City, IA 52242 USA; Department of Health Services Policy and Management, Arnold School of Public Health, University of South Carolina, Columbia, SC 29208 USA; Department of Health Management and Policy, College of Public Health, University of Iowa, Iowa City, IA 52242 USA; Department of Oncology (Medical), Mayo Clinic, Rochester, MN 55905 USA; Division of Hematology, Oncology and Blood and Marrow Transplantation, Department of Internal Medicine, Carver College of Medicine, University of Iowa, Iowa City, IA 52242 USA; Program in Pharmaceutical Socioeconomics, Department of Pharmacy Practice and Science, College of Pharmacy, University of Iowa, Iowa City, IA 52242 USA

**Keywords:** Geographic access to care, Cancer, Chemotherapy, Oncologists

## Abstract

**Background:**

An aging population, with its associated rise in cancer incidence and strain on the oncology workforce, will continue to motivate patients, healthcare providers and policy makers to better understand the existing and growing challenges of access to chemotherapy. Administrative data, and SEER-Medicare data in particular, have been used to assess patterns of healthcare utilization because of its rich information regarding patients, their treatments, and their providers. To create measures of geographic access to chemotherapy, patients and oncologists must first be identified. Others have noted that identifying chemotherapy providers from Medicare claims is not always straightforward, as providers may report multiple or incorrect specialties and/or practice in multiple locations. Although previous studies have found that specialty codes alone fail to identify all oncologists, none have assessed whether various methods of identifying chemotherapy providers and their locations affect estimates of geographic access to care.

**Methods:**

SEER-Medicare data was used to identify patients, physicians, and chemotherapy use in this population-based observational study. We compared two measures of geographic access to chemotherapy, local area density and distance to nearest provider, across two definitions of chemotherapy provider (identified by specialty codes or billing codes) and two definitions of chemotherapy service location (where chemotherapy services were proven to be or possibly available) using descriptive statistics. Access measures were mapped for three representative registries.

**Results:**

In our sample, 57.2 % of physicians who submitted chemotherapy claims reported a specialty of hematology/oncology or medical oncology. These physicians were associated with 91.0 % of the chemotherapy claims. When providers were identified through billing codes instead of specialty codes, an additional 50.0 % of beneficiaries (from 23.8 % to 35.7 %) resided in the same ZIP code as a chemotherapy provider. Beneficiaries were also 1.3 times closer to a provider, in terms of driving time. Our access measures did not differ significantly across definitions of service location.

**Conclusions:**

Measures of geographic access to care were sensitive to definitions of chemotherapy providers; far more providers were identified through billing codes than specialty codes. They were not sensitive to definitions of service locations, as providers, regardless of how they are identified, generally provided chemotherapy at each of their practice locations.

## Background

Patients who live in areas where chemotherapy providers are scarce must travel extended distances to receive treatment [1–3]. Research suggests that some patients who may benefit from chemotherapy remain untreated because of where they live [1, 2, 4–6]. Thus measures of geographic access to chemotherapy have been constructed to better understand these potential barriers to treatment. The two most common geographic access to care measures include local area density and distance to the nearest chemotherapy provider [1, 7–9]. To construct these measures, researchers must accurately identify chemotherapy providers and their service locations. Failure to account for all types of service locations can bias estimates of geographic access to chemotherapy. For example, in a recent study, the median driving time to the nearest chemotherapy provider decreased from 51.6 to 19.2 min when visiting consulting clinics were included [10].

Measures of geographic access to chemotherapy are often created using Medicare claims because they offer detailed information regarding treatments, providers and patients [11]. Unfortunately, even with this information, creating measures of geographic access to chemotherapy is not completely straightforward, as noted in the literature [12, 13]. Baldwin et al. state that researchers may need to distinguish between those who claim to be oncologists (through specialty codes) and those who do the work of an oncologist (through billing codes) when identifying chemotherapy providers [12]. They also acknowledge that identifying service locations is not trivial, as providers can and do practice at more than one location [12]. Should researchers assume that chemotherapy can only be provided at locations where it has been in the past, i.e. “proven” service locations? Or is reasonable to assume that chemotherapy may be available at each practice location, i.e. “possible” service locations?

Although the challenges of identifying providers and service locations have been previously noted, no research has quantified the effect of these various definitions on measures of geographic access to care. As there exists no standard protocol in how to define providers and locations, the definitions vary across studies. Some have used specialty codes to identify providers [14–16]. One paper used billing codes [17] and another used both billing and specialty codes [5]. Another strategy has been to supplement Medicare data with outside sources, such as the American Medical Association (AMA) physician masterfile or the Area Health Resource Files [1, 2, 4, 18–20]. Although doing so has been shown to capture more oncologists [12, 13], our objective was slightly different. We were interested in how various methods of identifying chemotherapy providers and locations impacted the two most commonly used measures of geographic access to chemotherapy: local area density and distance to nearest provider.

## Methods

### Data

We used retrospective data from the 2006 Surveillance, Epidemiology and End Results (SEER)-Medicare database. As a network of population-based cancer registries, SEER currently covers approximately 28 % of the U.S. population [21]. SEER-Medicare data is often used to study geographic access to chemotherapy for cancer patients because it offers detailed patient and provider information [11, 22].

Beneficiary ZIP code was abstracted from the SEER Patient Entitlement and Diagnosis Summary File (PEDSF) file. Medicare claims were used to identify chemotherapy use, provider specialty, and service location. We limited our analysis to the Carrier claims because only these report both provider specialty and location information. Driving times were calculated by Microsoft MapPoint. We chose driving-time distance instead of a straight Euclidian distance, i.e. “as the crow flies,” to account for topology (e.g. mountains, lakes, etc.) and their associated road structures, along with variations in travel time due to speed limits.

**Fig. 1:**
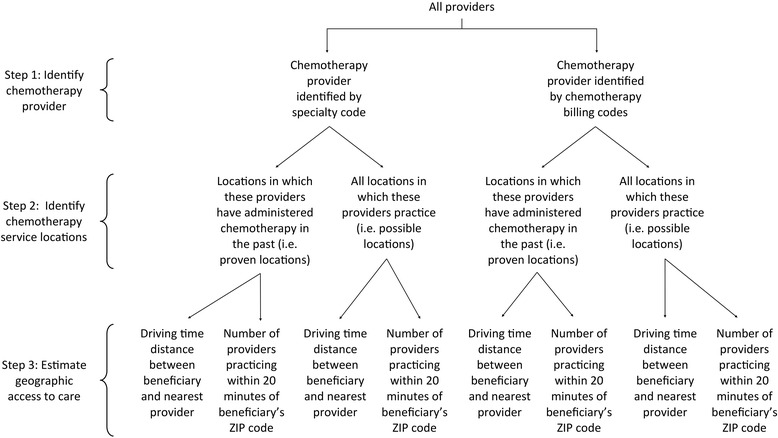
Algorithm for estimating measures of geographic access to chemotherapy. Two definitions of chemotherapy provider and two definitions of service locations are considered

To create our study cohort, we excluded unmatched ZIP codes. In 2006 35,206 Medicare beneficiaries across the 18 SEER registries were diagnosed with lung cancer. After excluding the beneficiaries for whom distances could not be calculated (*N* = 46) and whose residence ZIP code did not map onto a SEER registry according to MapPoint (*N* = 764), our final sample included 34,396 beneficiaries and their claims. Our methodology, which consisted of 2x2 possible ways of identifying providers and locations, is visually depicted (Fig. 1).

### Identifying chemotherapy providers

When we identified chemotherapy providers by specialty codes, individuals reporting a Hematology/Oncology (83) or Medical Oncology (90) specialty code on any claim were included. This would also apply to providers who reported multiple specialties among their claims. With this methodology, it is possible for an individual to *not* be labeled a chemotherapy provider even if they repeatedly billed for chemotherapy because an oncology specialty code was never reported. Chemotherapy providers were also identified through chemotherapy-related billing codes, Healthcare Common Procedure Coding System (HCPCS) codes and International Classification of Diseases, Clinical Modification diagnosis codes (ICD9-CM DX), provided by the SEER-Medicare program [23] and reported in Table 1. Hence an individual was considered a chemotherapy provider if they billed for chemotherapy-related services. With this method, it was possible for individuals who reported an oncology specialty code on claims to *not* be labeled an oncologist because none of their claims were chemotherapy related.

**Table 1:** Codes used to identify chemotherapy-related services in Medicare Carrier claims

Type of Code	Codes
HCPCS	96400-96549, J9000-J9999, Q0083-Q0085
ICD9-CM DX	V581, V662, V672

### Identifying chemotherapy service locations

Chemotherapy service locations were defined as either “possible” or “proven.” Proven service locations included only the practice locations from which providers billed Medicare for chemotherapy-related services. Possible service locations included all of a provider’s practice locations, regardless of whether a particular location ever filed a chemotherapy-related claim, under the assumption that each location *could* provide chemotherapy. The first method provides then a lower-bound estimate of service locations and the second an upper bound.

### Geographic access to chemotherapy measures

For a total of four possible specifications (specialty codes to identify provider and their proven service locations; specialty codes to identify provider and their possible service locations; billing codes to identify providers and their proven service locations; and billing codes to identify providers and their possible service locations), we estimated the two most commonly used measures of geographic access to chemotherapy: local area density and distance to nearest chemotherapy provider [24–26]. Measures of local area density form the basis for the increasingly popular floating catchment area methods and kernel density estimation methods [26]. In this paper, local area density was calculated by summing the number of unique chemotherapy providers within a 20-min driving-time radius from each beneficiary’s ZIP code centroid. Distance to nearest oncologist was calculated by the driving-time distance, in minutes, between centroids. If the beneficiary resided in the same ZIP code as the nearest chemotherapy service location, the distance between the two was set to zero. It is important to note that these measure *potential* access to chemotherapy and not actual utilization.

We calculated descriptive statistics and assessed differences between the estimates with Student’s t-tests. All analyses were conducted using SAS Software, version 9.3. Maps were created using Microsoft MapPoint 2013 for three representative SEER registries (Iowa, Connecticut and New Jersey) which vary in their degree of rurality.

## Results

Among the 216,438 unique providers with usable ZIP code information who cared for our study population in any capacity, 4,424 reported an oncology specialty code and 6,253 billed Medicare for a chemotherapy-related service. These 4,424 providers, as defined by specialty codes, provided chemotherapy in 5,076 locations (proven service locations) and practiced in a total of 5,157 locations (possible service locations). The 6,253 providers, as defined by chemotherapy billing codes, provided chemotherapy in 6,722 locations and practiced in 8,101 locations. Thus, on average, providers administered chemotherapy in 1.08-1.30 locations, depending on how provider and service locations were defined.

### Specialty codes and chemotherapy-related claims

Of the 6,253 unique providers who billed for chemotherapy-related services, 541 (8.7 %) reported more than one specialty and none were missing. The top four specialties reported by unique physicians included hematology/oncology (42.1 %), medical oncology (15.1 %), urology (9.8 %), and internal medicine (6.0 %). The two specialties of hematology/oncology and medical oncology comprised 57.2 % of the physicians.

However when we categorized the chemotherapy-related claims by specialty, we found that 91.0 % of the claims reported either a specialty of hematology/oncology (65.7 %) or medical oncology (25.3 %). The two next most commonly reported specialty on the claims were hematology (3.1 %) and internal medicine (2.9 %). This would indicate that, although self-reported oncologists are not the only ones administering chemotherapy, they are the ones who provide most of the observed chemotherapy-related services. We conducted an *ad hoc* analysis to confirm. Of physicians who reported only one specialty (*N* = 5,712), each hematologist/oncologist (medical oncologist) submitted on average 380.1 (388.8) chemotherapy claims during the study period. In contrast, each internist and urologist submitted on average 54.2, 10.0 claims, respectively.

### Geographic measures of access to chemotherapy

After characterizing the specialties associated with the claims, we estimated the two geographic access to care measures using the various provider and location definitions. We discuss results only for the proven locations, as access measures did not differ statistically across service location definitions.

### Specialty codes to identify chemotherapy provider

Identifying chemotherapy providers by specialty codes, we found on average 21.1 (SD = 29.8) providers within 20 min of a beneficiary, with a median value of 8 (Table 2). Almost 75 % of beneficiaries had access to at least one chemotherapy provider within 20 min. The driving-time distance to the nearest chemotherapy provider was on average 16.1 min (SD = 20.3). Half of the sample was within 10.7 min of a provider and patients in the 95^th^ percentile would need to drive 51.9 min to get to the nearest provider. These driving-time estimates are skewed towards zero because we are unable to assess distances within the same ZIP code. Thus, we also report the percent of patients that resided in the same ZIP code as a chemotherapy provider (Table 3). Overall, 23.8 % of beneficiaries resided in the same ZIP code as a chemotherapy provider, when defined by specialty code.

**Table 2:** Estimates of geographic access to chemotherapy by provider and service location definitions

Access to Chemotherapy Measure	Provider Identification Method	Proven Service locations		Possible Service locations	
		Mean (SD)	Median	Mean (SD)	Median
Number of chemotherapy providers within 20 minutes of beneficiary ZIP	Oncology Specialty Codes	21.1 (29.8)	8.0	21.3 (30.0)	8.0
	Chemotherapy Billing Codes	41.9 (52.3)	21.0	46.3 (57.3)	23.0
Driving-time distance to nearest chemotherapy provider (minutes)	Oncology Specialty Codes	16.1 (20.3)	10.7	15.9 (20.1)	10.6
	Chemotherapy Billing Codes	12.2 (16.6)	8.2	11.2 (15.9)	7.3

Data are from SEER-Medicare. Sample included all patients diagnosed with lung cancer that were reported to the SEER registries in 2006. Chemotherapy providers were identified in one of two ways: by specialty codes reported on Carrier claims (83 for Hematology/Oncology and 90 for Medical Oncology), and by chemotherapy-related billing codes on Carrier claims (Table 1). Chemotherapy service locations were identified in one of two ways: proven service locations are locations from where providers billed a chemotherapy-related service, and possible service locations are all locations in which the providers practice. Two geographic access to care measures are estimated: local area density (the number of chemotherapy providers within 20-minutes driving time of beneficiary ZIP code), and driving-time distance in minutes between the beneficiary ZIP code centroid and the centroid of the ZIP code of the nearest provider. These measures do not make any assumptions about whether beneficiaries received chemotherapy or from whom, and are instead a measure of potential access to care

**Table 3:** Percent of patients residing in the same ZIP code as a chemotherapy provider by provider definition for proven service locations

	Provider identified by specialty code	Provider identified by billing code	Absolute difference	*p*-value
All registries	23.8 %	35.7 %	11.9 %	<0.0001
Connecticut	23.5 %	36.0 %	12.4 %	<0.0001
Iowa	17.9 %	35.0 %	17.1 %	<0.0001
New Jersey	26.9 %	43.5 %	16.6 %	<0.0001

Data are from SEER-Medicare. Sample included all patients diagnosed with lung cancer that were reported to the SEER registries in 2006. Chemotherapy providers were identified in one of two ways: by specialty codes reported on Carrier claims (83 for Hematology/Oncology and 90 for Medical Oncology), and by chemotherapy-related billing codes on Carrier claims (Table 1). Proven service locations are locations from where providers billed a chemotherapy-related service, as opposed to possible locations, where providers billed for any service. These measures do not make any assumptions about whether beneficiaries received chemotherapy or from whom, and are instead a measure of potential access to care

### Billing codes to identify chemotherapy provider

We identified a substantially larger number of chemotherapy providers through chemotherapy billing codes. With this definition, beneficiaries had on average 41.9 (SD = 52.3) providers within 20 min, 2.0 times more than when identified by specialty codes (*p*-value < 0.0001). Driving time also decreased when identifying chemotherapy provider through billing codes. With this definition, beneficiaries were on average 12.2 (SD = 16.6) minutes away from a chemotherapy provider, 1.3 times closer than when identified by specialty codes (p-value < 0.0001). Over three quarters (79.4 %) had access to at least one chemotherapy provider within 20 min and over a third (35.7 %) resided in the same ZIP code as a chemotherapy provider, a 50.0 % increase compared to identifying providers through specialty code (*p* < 0.0001).

### Geographic variation in access to chemotherapy

**Fig. 2:**
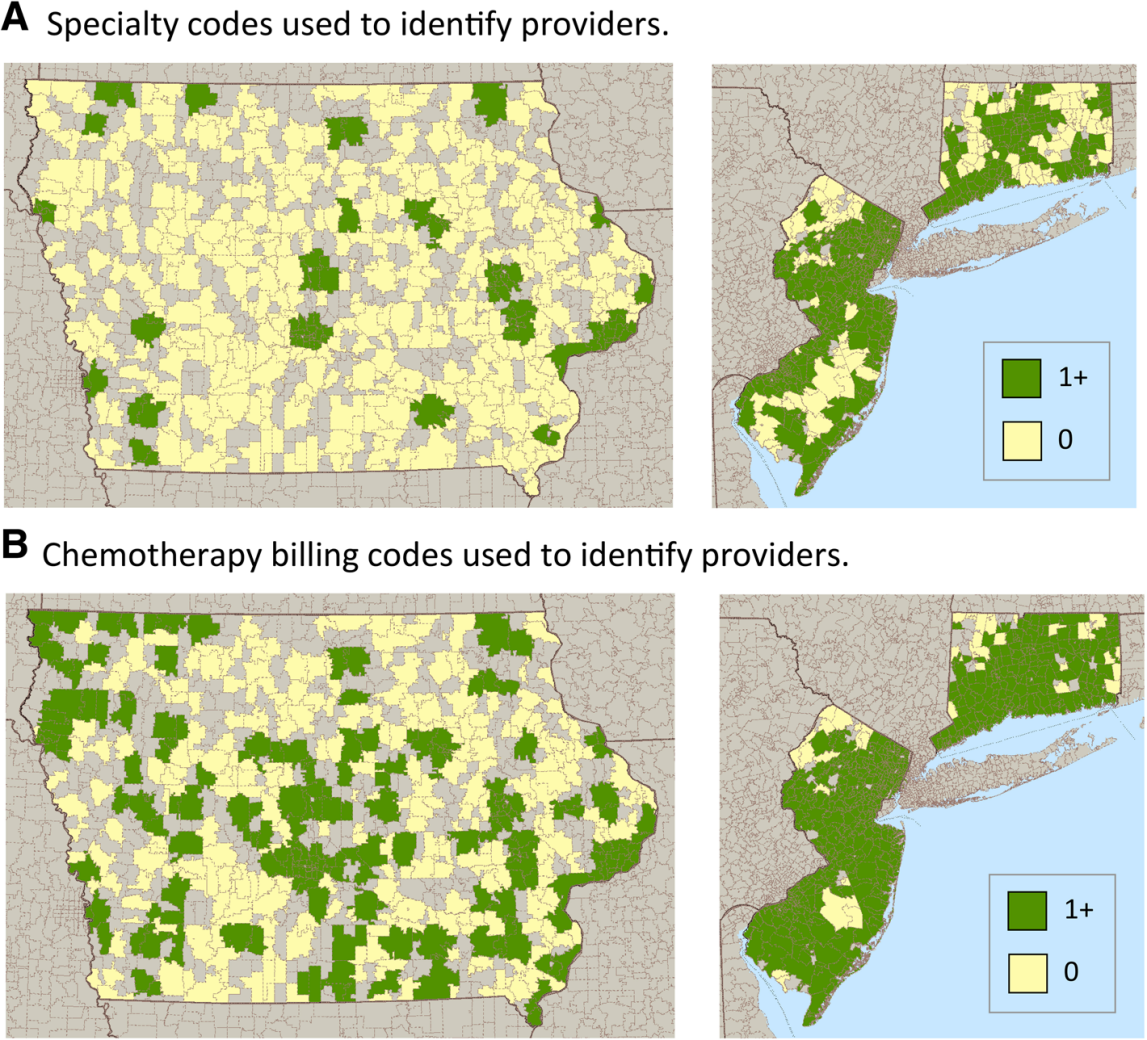
Access to chemotherapy providers within a 20-min driving-time radius for lung cancer patients. Three representative SEER registries are included: Iowa, New Jersey and Connecticut. In Panel **a**, providers were identified using specialty codes. In Panel **b**, providers were identified by chemotherapy-related billing codes. Brownish-grey areas indicate that there were no patients diagnosed with lung cancer in that ZIP code during the study period. Green ZIP codes indicate that a chemotherapy provider was available within 20-min driving time of that ZIP code. No chemotherapy provider was available within a 20-min driving time for yellow ZIP codes. Maps were created using Microsoft MapPoint 2013

Geographic access to chemotherapy (defined here as any provider practicing in their proven locations within 20 min of a beneficiary) was mapped for three representative SEER registries in Fig. 2. Panel A identified chemotherapy providers through specialty codes and panel B through chemotherapy billing codes. Brownish-grey areas signify that no beneficiary was diagnosed with lung-cancer in that ZIP code during the study period. Regardless of which method was used to identify providers, fewer Iowa beneficiaries were within a 20-min driving radius of a chemotherapy provider than beneficiaries residing in New Jersey or Connecticut. Similarly, Iowa beneficiaries were less likely to reside in the same ZIP code as a chemotherapy provider than beneficiaries residing in Connecticut or New Jersey: 17.9 % when identified by specialty code and proven locations in Iowa versus 23.5 % in Connecticut and 26.9 % in New Jersey (Table 3). When identified by chemotherapy billing codes, 35.0 % of Iowa beneficiaries resided in the same ZIP code as a provider, a substantial and statistically significant increase over identification by specialty code (*p* < 0.0001). Indeed, this rate was similar to the rate in Connecticut (36.0 %), although still less than New Jersey (43.5 %).

## Discussion

Access to treatments for cancer, whether geographic or financial, continues to be of interest to policy makers and researchers [1, 10, 27, 28]. However, previous papers estimating geographic access to chemotherapy using Medicare claims have not assessed whether their results are sensitive to how chemotherapy providers and service locations are identified. We found that the method of identifying who, but not where, chemotherapy is provided affected our two measures of geographic access to chemotherapy.

In our analysis, a number of specialties in addition to hematology/oncology and medical oncology billed Medicare for chemotherapy-related services, with 42.8 % of the providers associated with chemotherapy claims reporting a specialty other than hematology/oncology or medical oncology. Even so, physicians who reported a specialty of hematology/oncology or medical oncology provided the vast majority of the chemotherapy services (91.0 % of the claims).

When we identified chemotherapy providers through chemotherapy billing codes rather than specialty codes, we found that average driving-time distances for beneficiaries decreased by almost a quarter and the average number of providers within 20 min increased two-fold. An additional 50 % of beneficiaries resided in the same ZIP code as a chemotherapy provider when providers were identified using billing codes instead of specialty codes (35.7 % versus 23.8 %). This suggests that a number of oncologists might be reporting a specialty other than hematology/oncology and medical oncology. There are a number of reasons why this may be the case. First, all oncologists are trained in internal medicine before they specialize in oncology. Also, specialty certification is independent of maintaining one’s license, the former not mandatory for practice. Finally, accuracy in specialty code reporting is not required for reimbursement. Any/all of these factors could cause underreporting of oncology specialty codes.

The increase in geographic access to chemotherapy when providers are identified through chemotherapy billing codes instead of specialty codes is consistent with the literature showing underreporting of oncology specialty on Medicare claims [12, 13]. This does not necessarily imply that all of the providers associated with chemotherapy claims were indeed oncologists. It may also be the case that other specialties truly are providing chemotherapy. One could concoct a scenario where chemotherapy was provided by a non-oncologist under extenuating circumstances, but to which future provision of chemotherapy from that same provider is unlikely. Thus there is an additional dimension which has yet to be explored in the literature - whether past provision of chemotherapy is indicative of future access.

Although our measures of geographic access to chemotherapy varied substantially by how we identified chemotherapy providers, there was no statistical difference across service location definitions. This was due to the fact that chemotherapy providers in this sample in general provided chemotherapy at each location in which they practiced.

There are limitations to our study. First, we only utilize Medicare claims. Inclusion of additional data would affect the magnitude of our estimates, as others have found [13]. Unfortunately, NCI no longer releases unencrypted Unique Physician Identification Number (UPIN) or National Provider Identifier (NPI), rendering these linkages impossible. Our contribution to the literature, however, is not providing “the” estimate of geographic access to chemotherapy but instead assessing how definitions of “who” and “where” affect these estimates, something that others have noted might be an issue but never quantified [12]. In addition, we only include Carrier claims, which comprise about 75 % of chemotherapy claims. However the outpatient files do not include specialty information.

Another limitation is that we only include lung cancer patients and their providers. However, unless lung cancer patients are systematically avoiding some chemotherapy providers by how the providers report their specialty, our estimates of geographic access to care *across* definitions of providers and locations will not be biased. If some oncologists are sufficiently specialized (e.g. at an NCI-designated comprehensive cancer center) such that they never treat lung cancer patients, then they would not be captured in our analysis. However, these sorts of locations would also likely have oncologists that do specialize in lung cancer. Although absolute measures of access would not be accurate, relative measures across the various definitions, our outcome of interest, would still be valid. We also only include a single year of data. In doing so we may not capture physicians and their service locations if they bill Medicare less than once a year. Finally, our distances are calculated between ZIP code centroids. We acknowledge that distances between centroids are not as accurate as mailing addresses, and that driving times within a centroid is not zero. In addition, for ZIP codes that cover a large geographic area, the next closest ZIP code may be more than 20 min away. If there are no chemotherapy providers in the same ZIP code as the patient, that area would be considered as not having access to chemotherapy as we have defined it. ZIP codes can also change over time, increasing the complexity of geographic analyses.

## Conclusions

Improving geographic access to care and reducing health disparities have been of interest to healthcare providers and policy makers for some time and will continue to be so. Oncology services in particular are estimated to experience shortages in the coming years due to an aging population and insufficient growth in the physician workforce [29]. The effect is likely heterogeneous, as demographic compositions vary across areas and over time due to both aging in place and retirement migration. To the extent that measures of geographic access to care affect health policy, it is imperative that these measures be accurate. Our study suggests that measures of access to chemotherapy are more sensitive to how one identifies chemotherapy providers than service locations. Given that others have shown underreporting in specialty codes, perhaps both methods of identifying providers ought to be used to bound estimates of measures of geographic access to care.

## Abbreviations

HCPCS, Healthcare Common Procedure Coding System; ICD9-CM DX, International Classification of Diseases – 9^th^ edition, Clinical Modification, diagnosis code; NCI, National Cancer Institute; NPI, National Provider Identifier; PEDSF, Patient Entitlement and Diagnosis Summary File; SD, standard deviation; SEER, Surveillance, Epidemiology and End Results; UPIN, Unique Physician Identification Number; ZIP, Zone Improvement Plan
